# Mechanisms of Resistance to Macrolide Antibiotics among *Staphylococcus aureus*

**DOI:** 10.3390/antibiotics10111406

**Published:** 2021-11-17

**Authors:** Maria Miklasińska-Majdanik

**Affiliations:** Department of Microbiology and Virology, Faculty of Pharmaceutical Sciences in Sosnowiec, Medical University of Silesia in Katowice, Jagiellońska 4, 41-200 Sosnowiec, Poland; mmiklasinska@sum.edu.pl

**Keywords:** macrolides, resistance, *Staphylococcus aureus*

## Abstract

Methicillin resistant *Staphylococcus aureus* strains pose a serious treatment problem because of their multi-drug resistance (MDR). In staphylococcal strains, resistance to macrolides, lincosamides, and streptogramin B (MLS_B_) correlates with resistance to methicillin. The rapid transmission of *erm* genes responsible for MLS_B_ resistance has strongly limited the clinical application of traditional macrolides such as erythromycin. On the other hand, in the age of increasing insensitivity to antibiotics the idea of implementing a therapy based on older generation drugs brings hope that the spread of antibiotic resistance will be limited. A thorough understanding of the resistance mechanisms contributes to design of antibiotics that avoid bacterial insensitivity. This review highlights the mechanisms of action of macrolides and mechanism of resistance to these antibiotics among *Staphylococcus aureus*.

## 1. Introduction

The discovery of antibiotics revolutionized the treatment of bacterial infections. Unfortunately, the widespread and inappropriate use of antibacterial drugs in human and veterinary medicine, as well as food industry, has led to the selection and expansion of resistant bacteria and an increased treatment failure ratio [1]. In 2014, the World Health Organization (WHO) warned of the risk of a post-antibiotic era [2]. Three years later, the WHO published a list of 12 pathogens that pose threats to public health and for which the discovery of effective antibiotics is a priority with *Staphylococcus aureus (S. aureus)* being one the list [1,3]. *Staphylococcaceae* covers 40 species, including pathogenic and non-pathogenic bacteria. *S. aureus* is one of the most important species responsible for many infections due to its numerous virulence factors. There is a wide range of *S. aureus* infections ranging from skin or soft tissue infections to urinary, respiratory, and skeletal system diseases. *Staphylococcal* infections can also lead to sepsis, septic shock, or opportunistic infections [4,5,6]. It is therefore important to understand the mechanisms of staphylococcal resistance to successfully fight drug-resistant strains in the future [1].

**Figure 1:**
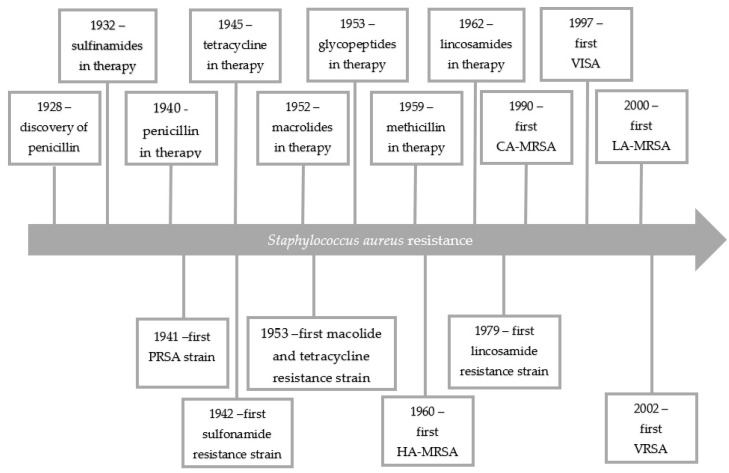
The development of resistance among *S. aureus* over the years [1,12]. PRSA—Penicillin Resistance *S. aureus*.

In 1928, Alexander Fleming discovered that mold inhibited the growth of staphylococci. The active ingredient was called penicillin [6]. Receiving the Nobel Prize for his discovery, Fleming warned against the development of penicillin resistance. Just as he predicted only 20 years after the discovery of penicillin the world was dominated by staphylococcal strains resistant to this antibiotic. In 1959, as a response to the growing penicillin resistance, semi-synthetic penicillins (e.g., methicillin), insensitive to penicillinases, were introduced into therapy. However, only a one year later, the first methicillin resistant *S. aureus* (MRSA) strain was isolated and 10 years later, MRSA was the cause of hospital epidemics (health-care-associated MRSA (HA-MRSA)) in the whole world. MRSA strains are developed through the acquisition by several *S. aureus* clones of the SCCmec chromosome cassettes by horizontal gene transfer [7]. In the 1990s, using molecular biology techniques, *S. aureus* was shown to spread in a hospital environmental in a clonal way. The presence of just two *S. aureus* strains increases the risk of horizontal gene transfer and spread of resistance genes or virulence determinants [8]. MRSA strains have evolved into multiple lineages and their occurrence are region depended. The new lineages disappear when they reach their peak and then they are replaced with new ones [9]. Due to the diversity of MRSA clones, these isolates show different sensitivity to antibiotics, which makes them a therapeutic challenge [10]. Nowadays, MRSA strains pose a global health problem because of their multi-drug resistance and they are endemic in many health-care facilities. What is more, MRSA strains are isolated outside the hospital environment (community-associated MRSA (CA-MRSA)) and are associated with livestock exposure (livestock-associated MRSA (LA-MRSA)) [7]. The rapid transmission of genes encoding resistance among *S. aureus* is also worrying, and significantly reduces therapeutic possibilities [11,12]. Until recently, vancomycin was the drug of choice for the treatment of MRSA infections. Unfortunately, the vancomycin intermediate-resistance *S. aureus* (VISA) has been noted. The first vancomycin-resistant *Enterococcus* (*E. faecium)* strain was reported in 1998. The appearance of vancomycin resistant enterococci strains raised concerns about effectiveness of vancomycin-base MRSA therapy. At the same time, the reduced susceptibility to teicoplanin among *S. aureus* strains was noted. The first vancomycin resistant *S. aureus* (VRSA) strain was reported in 2002 in United States. According to the Clinical and Laboratory Standards Institute (CLSI) recommendations, *S. aureus* is considered resistant to vancomycin when the minimal inhibitory concentration (MIC) value is >16 μg/mL, while the VISA strains have a MIC in the range from 4 to 8 μg/mL. Strains referred to as heterogeneous vancomycin intermediate-resistance *S. aureus* (hVISA) have MIC values of 1–2 μg/mL [12,13,14,15]. Therefore, the risk of lack of therapeutic options for patients suffering from staphylococcal infections is real. Figure 1 shows the development of resistance among *S. aureus* over the years.

The subsequent problem in staphylococcal infections is an increasing cross-resistance to macrolide, lincosamide, and streptogramin B antibiotics because of their extensive use against Gram-positive bacteria. Resistance to MLS_B_ antibiotics is determined by the expression of *erm* and *msr* genes. Nowadays, macrolide-resistant methicillin-resistant *S. aureus* (MAC-MRSA) is one of the most clinically important microorganisms due to the ability to rapidly acquire resistance and the limited therapeutic options associated with it [16]. MAC-MRSA strains are believed to be one of the most common causes for clinical infections. Infections of this etiology are associated with increased mortality rates and, consequently, prolonged hospitalization and increased costs of treatment [16,17]. More than 80% of MRSA strains show simultaneous resistance to MLS_B_ antibiotics, whereas in methicillin-sensitive *S. aureus* (MSSA) strains, the prevalence of MLS_B_ resistance is about 40%. The correlation of *S. aureus* resistance to methicillin and macrolides depends on many mechanisms, such as changing the target of the antibiotic action (*erm* genes) or active removal of macrolides from the bacterial cell (*msr* genes). The presence of the above mechanisms significantly contributes to the limitation of the therapeutic possibilities of MRSA infections [18,19,20]. Table 1 shows the frequency of resistance mechanisms to macrolide antibiotics (constitutive MLS_B_ (cMLSB), inductive MLS_B_ (iMLS_B_), and macrolide and streptogramin B (MS_B_)) in MRSA and MSSA, respectively. On the other hand, distribution of the *erm* genes in MRSA and MSSA is presented in Table 2. The data for the preparation of Table 1 and Table 2 was collected from the most important studies on the presence of MLS_B_ resistance determinants among MRSA and MSSA strains over the years. MRSA strains usually show constitutive resistance to MLS_B_ antibiotics which indicates their multi-drug resistance. Among MSSA strains, the difference in the prevalence of cMLS_B_ and iMLS_B_ resistance phenotype is small, but the iMLS_B_ phenotype is the most common. In both methicillin-resistant and methicillin-sensitive *S. aureus*, the MS_B_ phenotype is relatively rare. The MRSA strains show resistance to MLS_B_ antibiotics mostly determined by the presence of the *ermA* or *ermC* genes. On the other hand, among MSSA strains, the *ermC* followed by *ermB* gene is usually observed [16,20,21,22,23,24,25,26,27,28,29,30,31,32,33,34,35,36,37]. The research on the occurrence of *msr* genes among MRSA and MSSA is much less frequent. However, the available works prove that the *msrA* gene is incomparably more frequent than the *msrB* gene [23,25,27,28,29,32,36,37]. The presence of *erm* and *msr* genes and MLS_B_ resistance phenotypes largely depends on the location, which will be discussed later in this review.

Because of the high prevalence of MLS_B_ resistance found in MRSA isolates, the spread of antibiotic resistance among these microorganisms should be controlled. Moreover, since *S. aureus* has acquired resistance to many life-saving antibiotics, such as vancomycin, the notion that older and less used antibiotics such as macrolides are still effective in treating staphylococcal infections seems to be promising in inhibiting the development of new resistances [4]. Macrolides are broad-spectrum antibiotics often used as first-line drugs. The development of new macrolide antibiotics would give hope for effective therapies against drug-resistant strains. Studying the mechanisms that determine bacterial resistance to antibiotics is vital to understanding this process and significantly contributes to research into new antibiotics that can avoid these mechanisms. Therefore, discussed in this review are the mechanisms of resistance to macrolides in *S. aureus*, which contributed to the limitation of their use in therapy seems justified and important.

## 2. Macrolide Antibiotics

**Figure 2:**
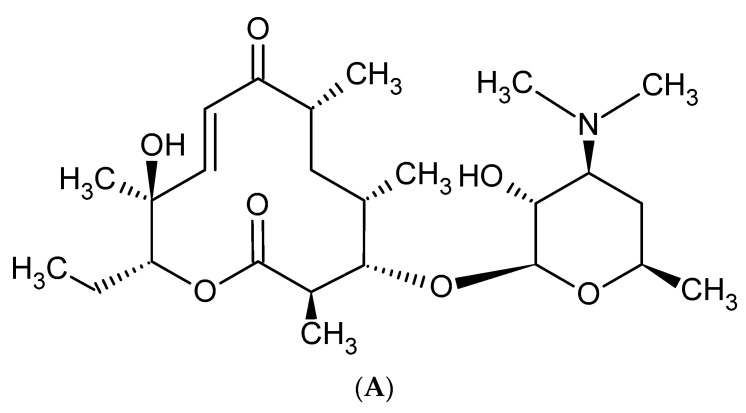

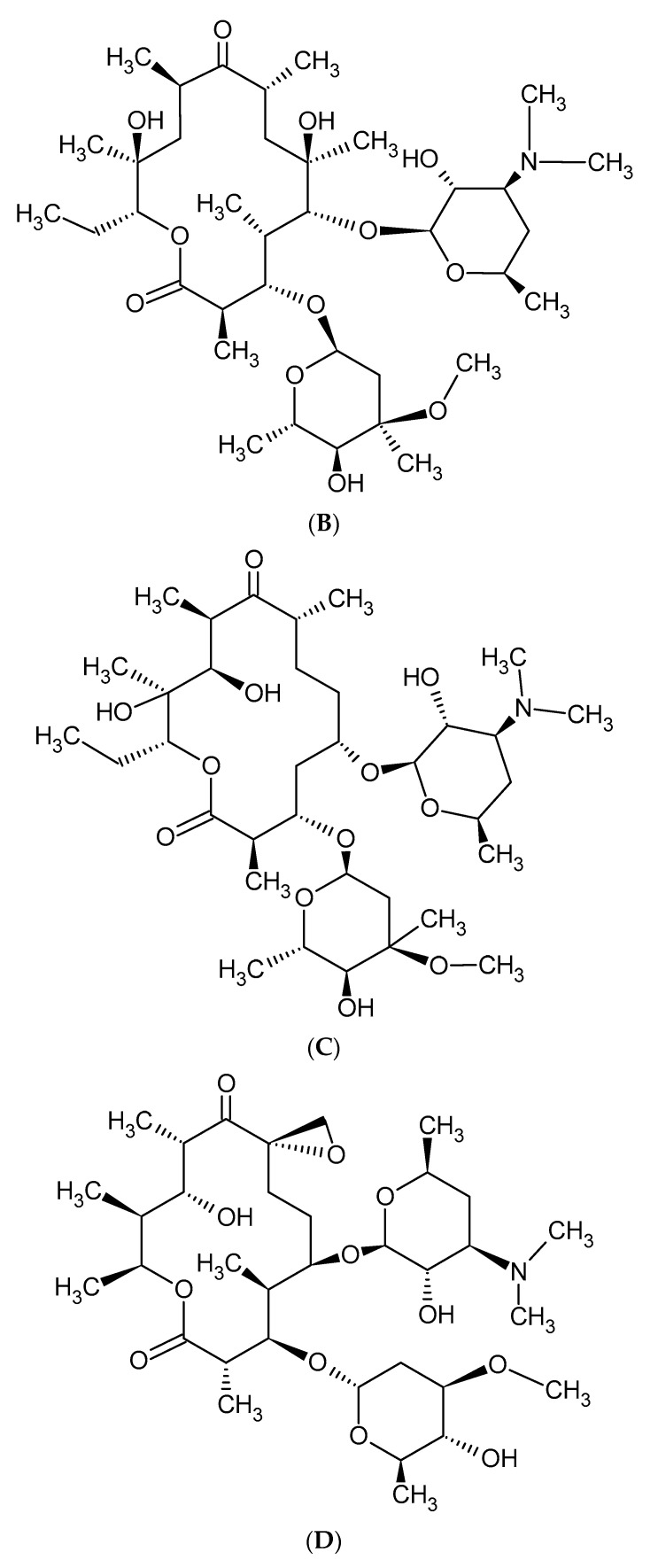

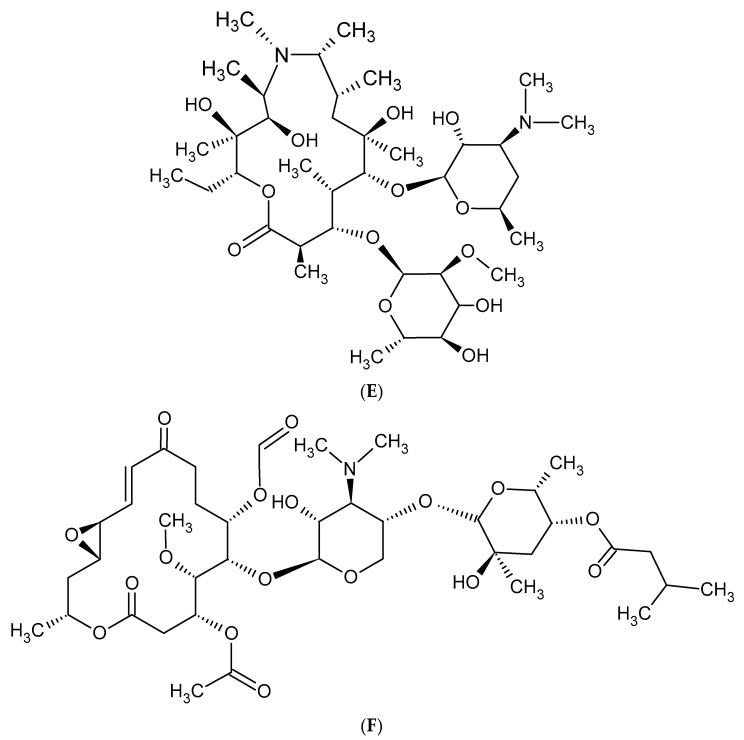
Methymycin (**A**), Erythromycin (**B**), Clarythromycin (**C**), Oleandromycin (**D**), Azithromycin (**E**), Carbomycin (**F**).

Macrolides are a group of protein synthesis inhibitors with broad spectrum activity. Macrolides are antibiotics of great clinical importance and are often used in therapy. The elementary component of macrolide molecules is the lactone ring consisting of 12, 14, 15, or 16 carbon atoms, combined with a deoxy sugar or amino sugar residues [19,38]. The structural formulas of the main macrolides are shown in the Figure 2.

Additionally, macrolide antibiotics can be divided according to their generation. The first—older—generation includes: erythromycin, carbomycin, spiramycin, oleandomycin, rosaramycin, and josamycin. Macrolides of the second, newer generation are semi-synthetic derivatives of natural products. The use of older macrolides is associated with many side effects, which were the main reason for the resignation from using these drugs and the case of synthesis the ‘side-effect-free’ macrolides, such as clarithromycin and azithromycin. The second-generation macrolides include: clartyromycin, azithromycin, midecamycin, dirythromycin, roxithromycin, flurithromycin, azithromycin, miokamycin, and rokitamycin [19,38]. The semi-synthetic drugs derived from erythromycin are characterized by better bioavailability and pharmacokinetics than natural macrolides, which allows reduction of the dosage. Azithromycin and clarithromycin have the greatest clinical applications [19]. Clarithromycin has methoxy group at position 6 of the erythromycin lactone ring (Figure 2C). In addition to reducing side effects during its use, clarithromycin also shows greater stability in the acidic environment of the stomach when compared to erythromycin. Moreover, it reaches a higher concentration in tissues than in serum. Azithromycin has nitrogen at the 9-position of its 15-membered lactone ring (Figure 2E). The undoubted advantages of azithromycin are its good tolerance and the ability to accumulate in tissues, as well as having a wider spectrum of action than erythromycin [19,39,40].

**Figure 3:**
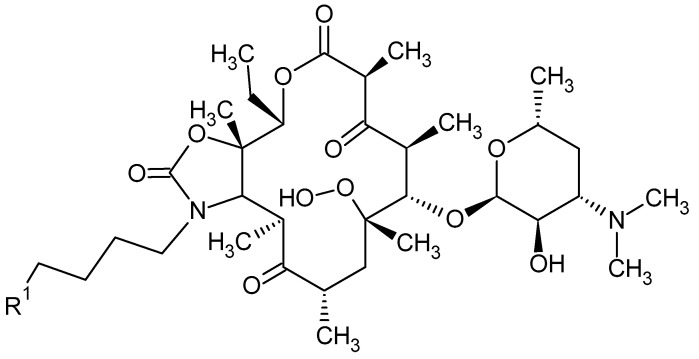
Telithromycin. R1—pyridylimidazole-alkyl side chain.

The introduction of second-generation macrolides into therapy improved the pharmacokinetics and pharmacodynamics of this group of antibiotics, but did not solve the problem of increasing antibiotic resistance. As a response to the growing insensitivity of microorganisms to antibacterial drugs, the third generation of macrolides—such as ketolide derivatives and telithromycin—emerged [19,41]. The antibiotics falling into the above class have a 3-keto group on the lactone ring instead of cladinose, which increases affinity to the ribosome 10- to 100-fold [42,43,44]. Currently, only a derivative of clarithromycin—telithromycin (Figure 3)—is used in the therapy. The Food and Drug Administration (FDA) has not approved the use of another ketolide—cethromycin because of insufficient activity in community acquired pneumonia treatment. Solithromycin is currently in its third phase of clinical trials [19]. Nowadays, research on the synthesis of new, safe, and highly effective macrolides is being conducted [45,46]. The recent reports on a new generation of macrolides suggest that they may be an effective therapeutic option [19].

Macrolide antibiotics have a broad spectrum of action. They are active against both Gram-positive and Gram-negative bacteria. Macrolides may slightly differ in the spectrum of action, but generally this range is similar. These differences are caused by the drug chemical structure influencing pharmacokinetic parameters [19].

**Figure 4:**
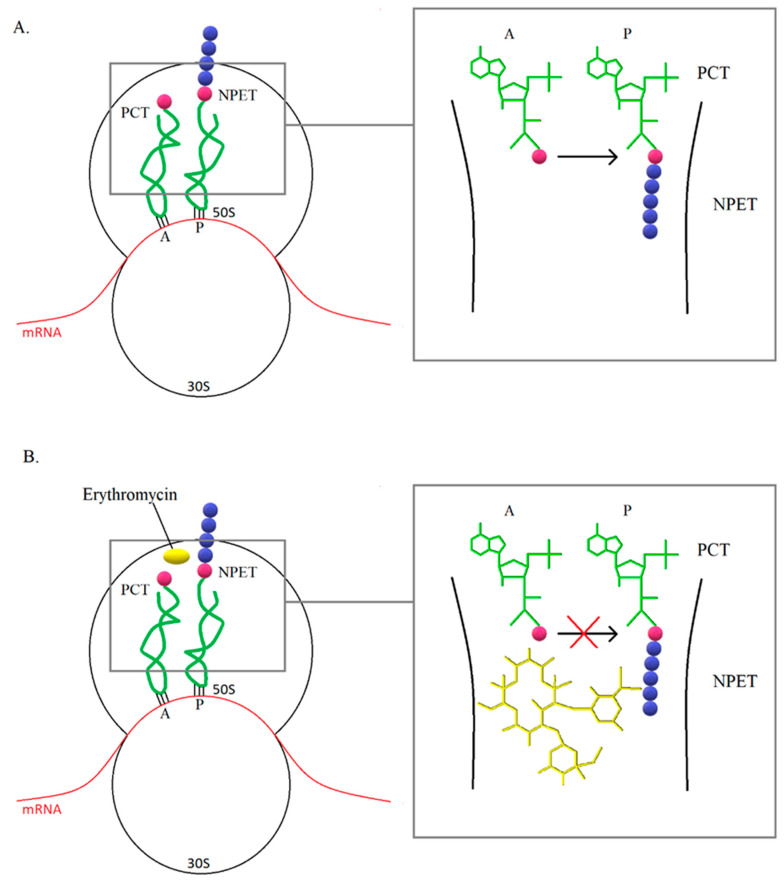
(**A**) The ribosome is a macromolecular complex that transcribes information encoded in mRNA into proteins. In prokaryotes, it consists of two subunits: the smaller (30S) and the larger (50S). The small subunit interacts with the mRNA and reads the genetic information. In turn, the large subunit is responsible for the formation of bonds between subsequent amino acids in the polypeptide chain. At the border of the subunits, there are a-site tRNA (**A**), e-site tRNA, and p-site tRNA (P) attachment sites responsible for translations. An extended polypeptide chain is linked to the P site, while further amino acids are delivered to the ribosome by aminoacyl-tRNA bound at the A site. The phenyl transferase center (PCT) is the center that catalyzes the reaction of polypeptide chain elongation. As each amino acid is added, the polypeptide chain is moved through the NPET. (**B**) The macrolide binding site is NPET near the PCT. For example, erythromycin (yellow), which when bound to the ribosome leads to the termination of the synthesis of the polypeptide chain. Binding of a macrolide antibiotic does not directly affect the protein synthesis process, but prevents further shifting of the polypeptide chain and translation is inhibited [47].

For a long time, mechanism of action of macrolides have been associated with inhibition of translation and thus inhibition of protein synthesis (Figure 4). In most cases, protein synthesis is interrupted in the presence of macrolides at the oligopeptide level of 5–11 amino acids. In the case of short peptides, there may be a cumulative ejection of the antibiotic from nascent peptide exit tunnel (NPET), which leads to the development of resistance. It has been observed that the synthesis of some proteins in bacteria treated with macrolide was at a comparable level with cells that were not affected by the antibiotic. In addition, some species have shown uninhibited protein synthesis in the presence of macrolide concentrations above the MIC value, therefore it is suggested that macrolides do not completely inhibit translation, but selectively stop protein synthesis. In addition, the mechanism of action of macrolides may be based on a change in the properties of the catalytic center of the ribosome, which leads to stopping translation or a change in the reading frame, resulting in abnormal synthesis of the polypeptide chain [47].

Today, macrolides are used mainly to combat MSSA infections. They are used in the treatment of upper respiratory tract infections, community-acquired pneumonia, sexually transmitted diseases, skin and gastrointestinal infections, and contagions caused by *Salmonella* spp. and *Shigella* spp. The macrolides are characterized by good penetration into the cells such as macrophages and granulocytes. Moreover, they reach high concentrations in cells and tissues. Therefore, macrolides take part in the processes of intracellular killing, thanks to which they exhibit antibacterial activity against microorganisms that can survive intracellularly, such as *S. aureus*, *Legionella* spp., *Mycobacterium* spp., *Listeria* spp. *Mycoplasma pneumoniae* (*M. pneumoniae*), *Haemophilus influenzae* (*H. influenzae*), and *Chlamydia* spp. [19,38]. Due to the selection of resistant strains during frequent macrolide therapy, the use of these drugs should be limited to cases where it is absolutely necessary [42,48].

## 3. Mechanisms of Resistance to Macrolide Antibiotics

Few years after the implementation of macrolides into the treatment, the first staphylococcal strains insensitive to these antibiotics appeared. Nowadays, the macrolide resistance is widespread worldwide and a large number of bacteria are resistant to MLS_B_ antibiotics [49]. The increasing insensitivity to macrolides among staphylococcal clinical strains is a consequence of their common use in the treatment of Gram-positive bacterial infections and is usually associated with resistance to lincosamides and streptogramins B [20].

The three main mechanisms of macrolide resistance in staphylococci are: (1) modification of the bacterial ribosome, (2) macrolide efflux from the bacterial cell/ribosome protection via ATP-binding-cassette family (ABC-F) proteins, and (3) enzymatic inactivation, but only the first two are important in the development of resistance in *S. aureus* [50]. Modification of the ribosomal target site causes a broad-spectrum resistance to macrolides, whereas efflux and enzymatic inactivation are of less importance. What is more, macrolides are characterized by a resistance mechanism showing different phenotypic expression which is essential in their interpretation [48]. The macrolide resistance genes are found on plasmids, transposons, and genomic islands and can be easily transferred horizontally between strains and species [50].

### 3.1. Modification of the Target Site of Macrolide Action

**Figure 5:**
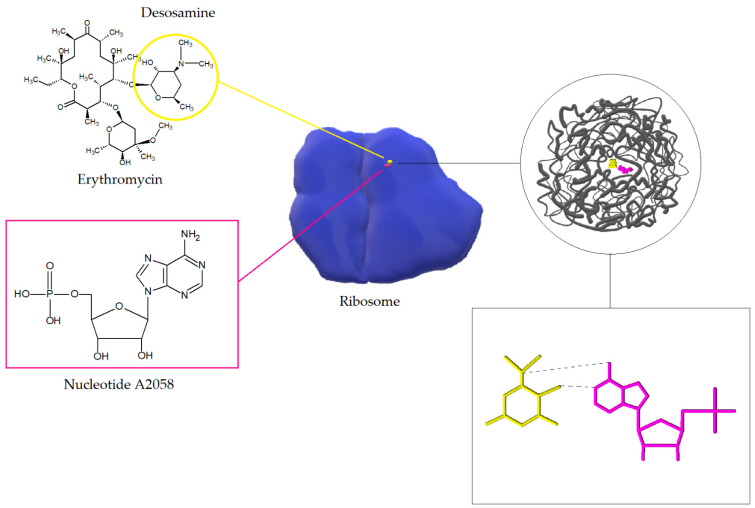
Interaction of macrolide (erythromycin) with 23S RNA nucleotide A2058. The figure shows the ribosome model (blue) and the model of the spatial arrangement of the exit tunnel (gray) with erythromycin (yellow) and nucleotides (pink) involved in the internalization of the antibiotic. After internalization of the macrolide with the binding site on the 50S ribosome subunit a hydrogen bond is formed between 2-OH group of desosamine and N1 in A2058 in NPET [52,53].

Macrolides, lincosamides, and streptogramins B affect the bacterial cell in the same way. The target sites of macrolides are nucleotides A2058 and A2059 located in the V region of the 23S rRNA domain of 50S ribosome and, rarely, nucleotide A752 located within domain II. The interactions of macrolides (on the example of erythromycin) with 23S RNA nucleotide A2058 is shown in the Figure 5. After internalization of the antibiotic, the protein exit channels in the 50S subunit are blocked, which leads to inhibition of polypeptide chain elongation. It is likely that macrolides and ketolides also disrupt the formation of new 50S ribosome subunits. Moreover, macrolides in high concentrations can also exert bactericidal activity [20,51].

**Figure 6:**
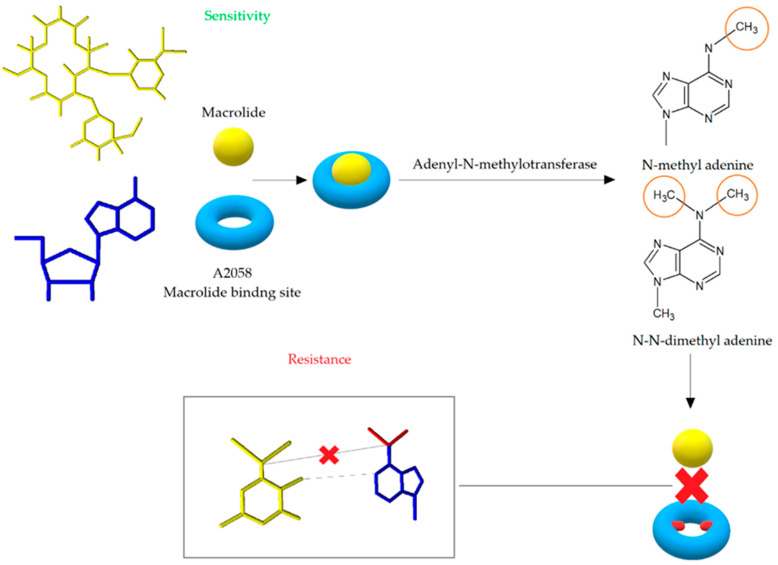
Modification of antibiotic’s binding site leading to cross resistance to macrolides, lincosamides, and streptogramin B. After the action of adentyl-N-methyltransferase, adenine is methylated, which prevents the binding of erythromycin to the binding site in the 50S ribosome subunit [52].

The modification of the antibiotic’s binding site leads to high-level and most commonly noted mechanism of resistance to MLS_B_ antibiotics. The change of target site is mediated by the adenyl-N-methyltransferase erythromycin resistance methylase (Erm) enzymes encoded by *erm* genes. The Erm enzymes are responsible for the methylation of adenine, which results in the formation of N-methyl adenine or N, N-dimethyl adenine and consequently post-transcriptional modification of the 23S rRNA structure [19]. The process of monomethylation or dimethylation depends on the type of the Erm enzyme, while it is not known whether dimethylation occurs in two steps [54]. Adenine methylation prevents macrolides from binding to their target site on the bacterial ribosome (Figure 6). Because MLS_B_ antibiotics share a common binding site, cross-resistance is thereby created [55]. The *erm* genes encoding methylase are localized on the high- and low-copy plasmids or transposons [19,56]. Forty *erm* genes divided into 14 classes have been recorded so far, but only the classes *ermA*, *ermB*, and *ermC* are important in the development of MLS_B_ resistance in *S. aureus* [42,50].

The expression of MLS_B_ resistance can be constitutive or inductive. In the first case, the synthesis of methylase with the participation of active mRNA occurs in a continuous and steady (constitutive) way. This leads to the development of resistance to all MLS_B_ antibiotics. In the case of inductive resistance, an inactive mRNA is formed and its activation requires the presence of an inducer, then the synthesis of methylase can start. Inactive mRNA has a hairpin structure consisting of the sequence coding a leader peptide and inverted repeats. As a result of contact with the inducer, the hairpin structure is destabilized during the translation of the leader peptide and the Erm methylase is translated. The whole sequence of *erm* gene is essential for the development of both types of resistances [19,48,57,58,59]. The induction is determined by the presence of attenuators upstream of the *erm* gene. The attenuators show differences in length or leader peptides. Bacterial strains with inductive resistance phenotype are insensitive only to antibiotics that induce methylase synthesis, i.e., macrolides with a 14- or 15-carbon lactone ring with sugar at C3 [19,59]. Resistance to 16-membered ring macrolides, lincosamides, and streptogramin B occurs only in the presence of 14- or 15-carbon ring macrolides as inducers [20,55,57,58].

**Figure 7:**
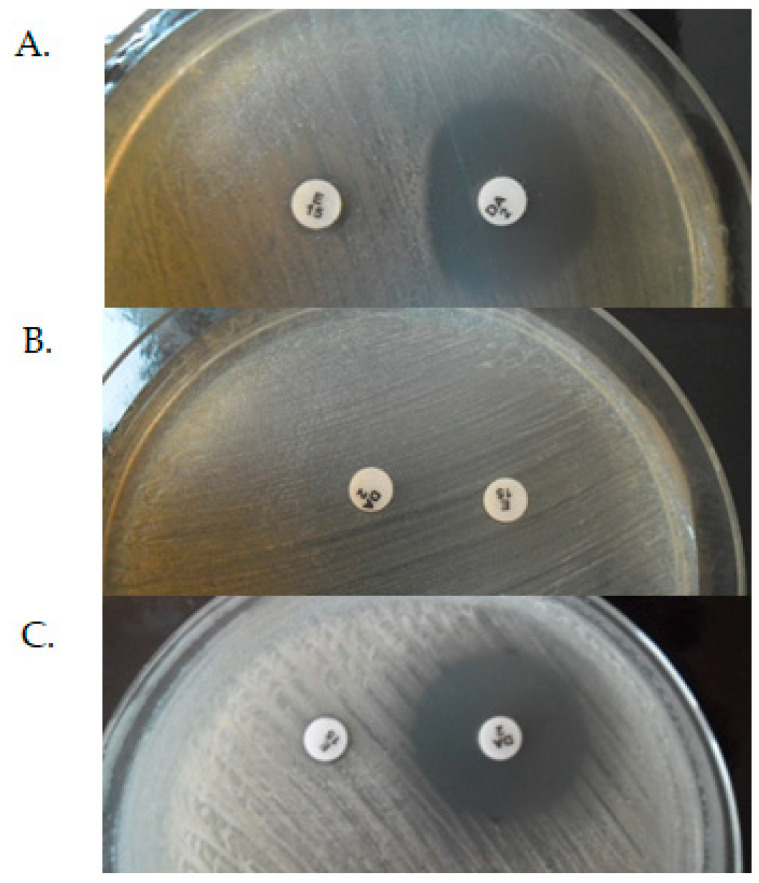
Inductive (**A**), constitutive (**B**), and MS_B_ (**C**) phenotypes of MLS_B_ resistance as determined by D-test (E-erythromycin, DA-clindamycin). The photos come from the author’s private archive.

The constitutive, inductive, and MS_B_ phenotypes of MLS_B_ resistance can be distinguished by D-test method, where a colony suspension equivalent to 0.5 McFarland unit is inoculated to Mueller–Hinton Agar with a 15 μg clindamycin and 2 μg erythromycin disks. According to European Committee on Antimicrobial Susceptibility Testing (EUCAST) recommendation, the distance between the edges of disks should be 12–20 mm. The zone diameter size and shape are interpreted after 18 h of incubation at 35 °C. In iMLS_B_ phenotype the diffusion of erythromycin in the agar leads to the characteristic flattening of the growth inhibition zone around the clindamycin disc on the side of the erythromycin disc (D-shaped)—Figure 7A. In cMLS_B_ phenotype (Figure 7B), the tested strain is resistant to both erythromycin and clindamycin, while in the case of the MS_B_ phenotype (Figure 7C), it is resistant to erythromycin and sensitive to clindamycin. In both of the above cases, there were no changes in the shape of the growth inhibition zones [20,57,58,60]. According to the EUCAST recommendations, the inductive resistance in staphylococci should be determined using a disc diffusion test due to the possible development of resistance to lincosamide—clindamycin during therapy despite phenotypic susceptibility to this antibiotic [60].

Since ketolides have a stronger affinity for the 50S ribosome subunit, they often show activity against strains with inductive *erm* genes. However, it is believed that dimethylation can determine ketolide resistance [51,61]. Clindamycin is not an inducer, but its use in the treatment of infections caused by inducible (iMLS_B_) strains may result in developing resistance in vitro. The selection of strains resistant to clindamycin during the treatment depends on factors such as type of infection, frequency of mutation, and size of bacterial inoculum. In the case of infections with high bacterial inoculums, such as pneumonia or extensive skin infections, the risk of developing constitutively resistant mutants increases [55,62,63,64]. Therefore, it is essential to correctly interpret both phenotypes of resistance. Macrolides, lincosamides, and streptogramins B should not be used in therapy for both the constitutive and inductive resistance phenotypes. Strains with the above resistance phenotypes should be treated as susceptible to streptogramins A. However, it must be noted that streptogramins lose their bactericidal effect towards strains with MLS_B_ resistance in favor of the bacteriostatic one [60].

The expression of the *ermA* and *ermC* genes is the most common cause for development of inductive resistance to MLS_B_ antibiotics in staphylococci [55]. The *ermB* gene (located on the Tn551 transposon) is present mainly in streptococci and enterococci [48]. Table 3 shows the most important studies on the frequency of *erm* and *msr* genes in *S. aureus* strains with the MLS_B_ resistance mechanism over the years. The data collected in the table confirm that the *ermC* gene is the most common determinant of MLS_B_ resistance, followed by *ermA* and *ermB* genes. The *msrA* gene is less frequent, while the *msrB* gene is found rarely. The most common determinant of cMLS_B_ and iMLS_B_ resistance is *ermC* gene, followed by *ermA* gene.

**Figure 8:**
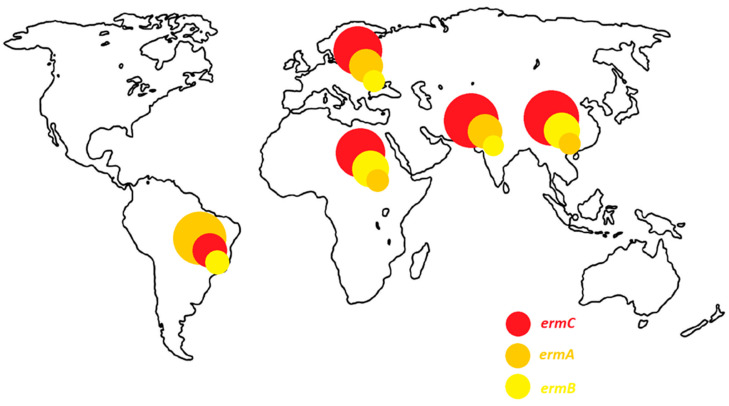
Prevalence of *erm* genes in the world.

Distribution of genes determining resistance to macrolide antibiotics depends on location of the study. The study on the *erm* and *msr* genes is widely conducted in the Middle East. In this region, the resistance to macrolide antibiotics (cMLS_B_, iMLS_B_ and MS_B_) is most often determined by the presence of the *ermC*, followed by *ermA* gene. Interestingly, the *ermB* gene is isolated more frequently in China and Egypt than in other regions of the world. It is also a common determinant of constitutive-type resistance in these areas. On the other hand, in South America, the *ermA* gene is the most frequent. It is isolated as often from strains with a constitutive as well as an inductive resistance phenotype. In Europe, the dominant gene is the *ermC*, but the *ermA* gene is isolated with a comparable frequency. The distribution of *erm* genes depending on the region of the world is presented in the Figure 8.

Other important factors influencing the prevalence of resistance genes are local resistance mechanisms, the presence of other resistance genes, the study group, or years of study. All studies listed in the Table 3 were conducted on a large number of clinical *S. aureus* strains. Most of them are prospective research. Since each study group consisted of a different number of strains, and these strains differed in their resistance mechanisms, it is difficult to develop a good tool to track the spread of *erm* genes over the years. However, when analyzing the data presented in the Table 3, it can be concluded that the frequency of genes, especially *ermA* and *ermC*, increases with time [4,16,20,21,22,23,24,25,26,27,28,29,30,31,32,33,34,36,37,65,66,67,68,69,70,71].

**Table 3:** Distribution of *erm* genes among MLS_B_-resistant *S. aureus* strains in various studies over the years.

MLS_B_ Antibiotic Resistance Phenotype	The Frequency of *erm* Genes (%)									Localization	Years	References
	*ermA*	*ermB*	*ermC*	*ermA/ermC*	*ermA/ermB*	*ermB/ermC*	*ermA/ermB/ermC*	*msrA*	*msrB*			
cMLSB	14.89	11.36	74.46	-	-	-	-	0	0	Iran		[23]
iMLSB	-	20.7	-	-	-	-	-	51.7	20.7		2018–2019	[23]
MSB	-	10	-	-	-	-	-	30	20			[23]
iMLSB	15.6	3.1	18.7	4.6	0	0	-	-	-	Nepal	2018	[22]
iMLS_B_	3.03	3.03	21.21	15.15	9.09	21.21	27.27	-	-	Jordan	2017	[33]
cMLSB	25.9	18.5	44.4	22.2	3.7	0	14.8	0	0	Iran	2016–2017	[65]
iMLSB	54.5	63.6	81.8	0	0	0	36.4	0	0			[65]
MSB	12.5	0	37.5	0	0	0	0	0	0			[65]
not distinguished	46.7	-	36.7	-	-	-	-	38.3	-	Iran	May 2017	[25]
not distinguished	8.6	-	22.9	37.1	-	-	-	5.7	-	Iran	2015–2016	[27]
cMLSB	13	-	19	17	-	-	-	1	0	Iran	2016	[28]
iMLSB	10	-	50	0	-	-	-	10	0			[28]
MS_B_	0	-	33	11	-	-	-	0	0			[28]
not distinguished	11	3.5	20.5	-	-	-	-	10.5	10.5	Iran	2014–2015	[66]
not distinguished	40.6	0	17.7	-	-	-	-	0	0	Iran	2014	[30]
iMLSB	11.1	22.2	44.4	-	-	-	-	-	-	Iran	2010–2012	[67]
not distinguished	21.6	38.7	90.1	-	-	-	-	-	-	China	2013–2019	[34]
iMLSB	30.5	1	69	-	-	-	-	-	-	China	2013–2016	[20]
cMLSB	69.5	99	31	-	-	-	-	-	-			[20]
not distinguished	59.4	8.1	24.3	16.3	5.4	2.7	2.7	-	-	China	2013–2015	[26]
not distinguished	3.7	22.2	40.7	3.7	-	3.7	-	0	0	China	2010–2011	[29]
cMLSB	16.66	54.16	0	25	0	4.16	0	-	-	China	-	[68]
iMLSB	0	0	100	0	0	0	0	-	-			
not distinguished	39.7	0	8.7	0.6	1.2	0	1.2	6.2	1.2	Brazil	2014–2019	[32]
cMLS_B_ + iMLS_B_	9.1	0	38.6	2.3	-	-	-	-	-	Brazil	2012	[31]
cMLSB	90	2.5	7.5	0	0	-	0	-	-	Brazil	2010	[69]
iMLSB	66.6	0	33.3	0	0	-	0	-	-			[69]
MSB	0	0	0	0	0	-	0	-	-			[69]
not distinguished	53.8	0	30.8	7.7	0	2.6	-	5.1	0	Brazil	2004- 2009	[37]
cMLSBiMLSB	9.37	-	46.87	6.25	-	-	-	0	-			[16]
iMLS_B_	17.6	-	29.41	29.41	-	-	-	0	-	Egypt	2021	[16]
MS_B_	-	-	-	-	-	-	-	100	-			[16]
not distinguished	30.3	85	99	0	34	85.6	22.9	-	-	Egypt	2018–2019	[24]
not distinguished	15.4	-	61.5	-	-	-	-	23.1	-	Italy	2013–2016	[70]
cMLSB	53.5	0.7	2.8	-	-	-	-	-	-	France	1995	[21]
iMLSB	9.8	0	22.2	-	-	-	-					[21]
not distinguished	0	0	29.9	-	-	-	-	-	-	Austria	2004–2008	[71]
not distinguished	56.85	0	25.38	1.52	0	0	0	5.15	0	Belgium	2008	[36]
cMLS_B_	-	-	28.9	-	-	-	-	-	-			[4]
iMLS_B_	25.5	-	29.4	-	-	-	-	-	-	Serbia	2016	[4]
MS_B_	0	0	0	0	0	0	0	95.6	95.6			[4]

-: no data, cMLS_B_: constitutive phenotype of resistance to macrolides, lincosamides and streptogramin B. iMLSB: inductive phenotype of resistance to macrolides, lincosamides, and streptogramin B. MS_B_: resistance to 14- and 15-membered macrolides and streptogramin B.

Constitutive variants associated with the *ermA* genes occur not so often, while the development of constitutive resistance from inductive *ermC* genes is much more frequent. Strains with constitutive resistance are caused by deletions, duplications, insertions, and rarely point mutations. These mutations are usually located up to the 5′ end of the *erm* gene. The various number of the *ermA* and *ermC* gene copies and their different locations in cell are likely cause of differences in the frequency of cMLS_B_ strains [72]. The *ermA* gene in *S. aureus* is usually located on the Tn554 transposon (att554 site of staphylococcal chromosome), which is carried by a conjugative plasmid pWBG4. Tn554 transposone contains three transposase genes (*tnpA*, *tnpB*, and *tnpC*), the *spc* gene, the discussed *ermA* gene and open reading frame (ORF). Importantly, the Tn554 transposon was found to be integrated in the J2 region of the SCCmec cassette. On the other hand, the *ermC* gene is carried by three different plasmids: pNE131, pE194, and pSES22 [48,50]. However, pE194 plasmid is not common among staphylococci. The *erm* (*33*) gene, found in *Staphylococcus sciuri* (*S. sciuri*), is a hybrid between the *ermA* and *ermB* genes resulting from recombination. The *ermA* or *ermC* genes usually present in MRSA strains, while *ermC* gene causes MLS_B_ resistance in MSSA isolates as mentioned before [40,50,72,73]. The *ermB* gene is located on the Tn551 transposon. Transposon Tn551 consists of the *ermB*, *tnpR* and *tnpA* genes and is sometimes present in CA-MRSA DNA or on plasmids containing the *blaZ* operon. Transposons can translocate from chromosome to plasmid and vice versa. If they are located on a conjugation plasmid, they can move from cell to cell, leading to the transfer of resistance genes [50].

**Figure 9:**
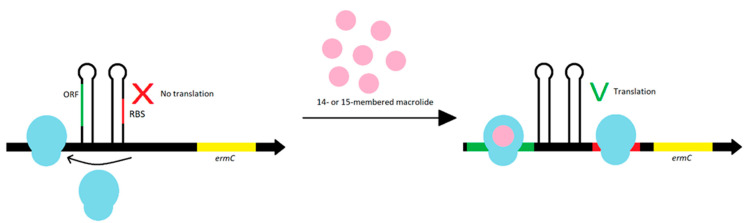
The ORF at the start codon synthesizes leader peptides. The ORF translation leads to the formation of stamped structures, which prevents the ribosome from binding to RBS. In the presence of an inducer, translation of the leader peptides is inhibited, with the ribosome binding to RBS, and translational of resistance genes may occur.

The *ermA* and *ermC* gene expression is regulated during translation. The *ermA* gene is preceded by the pepL and pep1 genes, which produce two leader peptides. The *ermC* gene is preceded only by the *pep* gene [72]. The stapled structures of *pep* gene inhibit ribosome access to ribosome binding site (RBS) for the *erm* gene. To Erm methylase synthesis, the induction is necessary—the prior internalization of the ribosome with macrolide antibiotics with a 14- or 15-membered lactone ring blocks translation of the leader peptide. The staple structure of the mRNA becomes destabilized, which allows the ribosomes to enter the RBS for the *erm* gene and a translation may occur. The ORF on the start codon of methyltransferase synthesizes leader peptides. Each peptide contains a macrolide stall motif. As a result of stopping the ‘ribosome-macrolide’ complex formation, the attenuator including RBS is interrupted, leading to the synthesis of methyltransferase (Figure 9). Most *erm* genes are expressed in inducible way because methylation of the ribosome negatively affects translation of bacterial peptides. Therefore, conditional resistance is more beneficial for bacteria [42,72,74].

### 3.2. Resistance Mechanism Related to msr Genes

The products of *msr* genes determine another mechanism of resistance to macrolide antibiotics, which is manifested by resistance to macrolides and streptogramin B (MS_B_ phenotype). The *msrA* gene was first identified on the pUL5050 plasmid in *Staphylococcus epidermidis* (*S. epidermidis*) strain, while the *msrB* gene has been found in pCH200 plasmid of *Staphylococcus xylosus* (*S. xylosus*) [21,49]. These genes form one class. The *msr* genes encode ATP binding cassette (ABC) transporters [42]. The development of resistance associated with *msr* genes is highly controversial. Until recently, the encoded proteins were thought to act as efflux pump. However, it is currently reported that they could play the role of protective proteins, which, by being internalized with the ribosome, cause the macrolide to be detached [49,51,75,76].

Expression of *msrA* is regulated, same as *erm* genes, via translation attenuation process. However, this mechanism needs higher level of inducer [42]. Constitutive resistance to macrolides and streptogramin B is associated with 320-bp deletion of the control region of *msrA*. Synthesis of MsrSA proteins, showing 98% homology to MsrA, also leads to MS_B_ type of resistance [77].

There are four main classes of Msr proteins: A, C, D, and E, which show 80% amino acid homology with each other. The role of Msr proteins is to displace the antibiotic from the ribosome [19,42]. The expression product of the *msrA* gene is the 488-aa ABC-F protein [50]. The MsrA protein belongs to class 2 of ABC-transporters. Based on phylogenetic analysis, ABC transporters have been divided into three classes. In contrast to other proteins from the ABC family, MsrA does not have a transmembrane domain in its structure [42]. Biologically active proteins of the ABC-transporter family contain four domains in their structure. Two of them are hydrophilic nucleotide binding domains (NBD) and another two are hydrophobic transmembrane domains (TMD). Hydrophobic domains, also known as membrane spanning domains (MSD), are made up of six transmembrane α-helices and form homo- and heterodimers. They are believed to determine the specificity of the substrates transported by ABC proteins [78].

**Figure 10:**
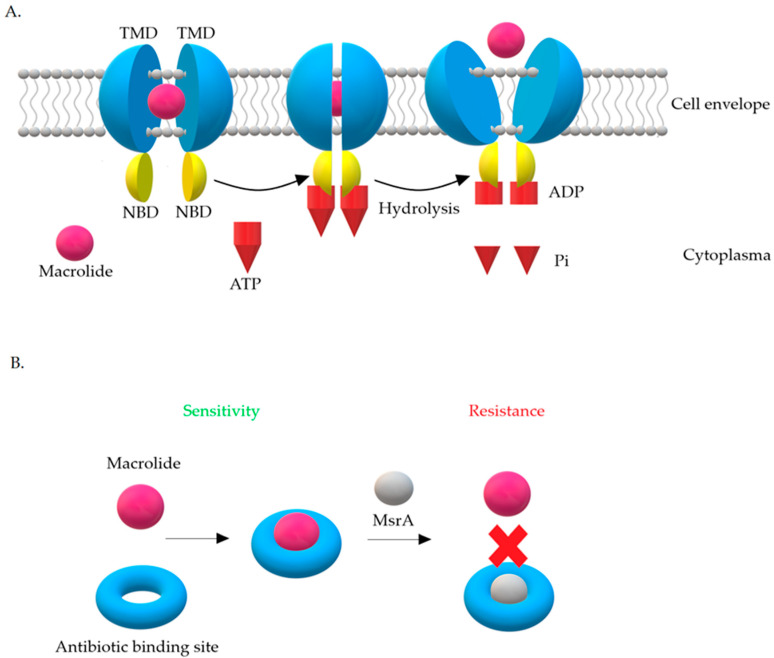
Mechanisms of resistance associated with the MsrA protein leading to resistance to 14- and 15-membered macrolides and streptogramin B (MS_B_ phenotype): (**A**) Macrolide efflux from the bacterial cell [80], (**B**) MsrA acts as a protective protein.

There are two hypotheses explaining the resistance mechanism associated with the MsrA protein. The first one assumes that it has ATP-dependent efflux pump activity, while according to the second one, MsrA acts as a protective protein (Figure 10) [42,76].

The efflux hypothesis suggests that active removal of the drug occurs with the participation of energy generated from ATP hydrolysis. NBD domains connect to ATP on the cytoplasmic side, constantly cooperating with TMD domains. Briefly, NBD domains act as an ‘engine’ that provides energy for substrate transport. Internalization with ATP and its hydrolysis lead to changes in the structure of proteins. These changes remove the drug from the cell. Transporters work on the principle of a specific pump, the role of which is to remove 14- and 15-membered macrolides and streptogramins B from the cell, preventing them from reaching their target site on the ribosome [76].

The hypothesis about proteins, which are ribosomal protectors, assumes that MsrA influences translation process by binding to the ribosome. This internalization leads to blockades of the antibiotic binding site on the 23S rRNA subunit, which is common to macrolides and streptogramins B [42,79]. This hypothesis has not been confirmed so far. What is more, it has been observed that, in staphylococci possessing the MsrA protein, less accumulation of erythromycin in the cell is seen. It is therefore highly probable that this protein interacts with a protein equipped with transmembrane domain necessary for drug elimination from the cell. Moreover, it has been shown that the activity of *msrA* is inhibited by the Gram-positive bacteria pump inhibitors—arsenate, dinitrophenol, or carbonyl cyanide m-chlorophenyl hydrazone—which supports the hypothesis of active removal of macrolides from the cell. However, *msrA* activity was not inhibited by the Gram-positive bacteria pump inhibitor with different mechanism of action—reserpine [77,79].

Regardless of the mechanism of action, it is known that the presence of *msrA* family genes is associated with resistance to 14- and 15- membered macrolides and streptogramin B (MS_B_ phenotype) and low-level resistance to ketolides [42]. The MS_B_ phenotype is usually determined by the presence of the *msrA* or *ermC* genes (Table 3). However, among the strains with this phenotype, the isolation of *msr* and *erm* genes is much less frequent in cMLS_B_ and iMLS_B_ phenotypes. Moreover, in contrast to the MLS_B_ resistance phenotype, there is no cross-resistance to 16-membered ring macrolides and lincosamides (even after induction) in MS_B_ phenotype and the effectiveness of therapy in this case can be achieved using 16-membered lactone ring macrolides, clindamycin, and streptogramin A. The MS_B_ type of resistance is regulated inductively. The inducers are 14- and 15-membered macrolides and resistance to streptogramins B occurs only in their presence [48]. The degree of MS_B_ resistance is independent of the number of *msrA* copies in the chromosome. Multiple copies of this gene do not increase the MIC values for erythromycin and streptogramins B and the introduction of a single copy of the *msrA* gene into the chromosome resulted in the same level of erythromycin resistance (MIC 70–80 mg/L). The MS_B_ resistance phenotype is determined using the double disks test with erythromycin and clindamycin [60,77].

### 3.3. Enzymatic Inactivation of Macrolides

**Figure 11:**
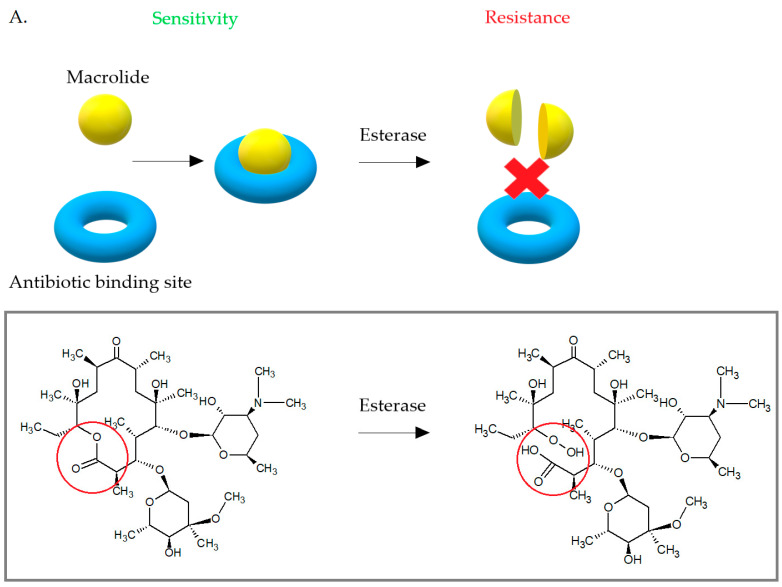

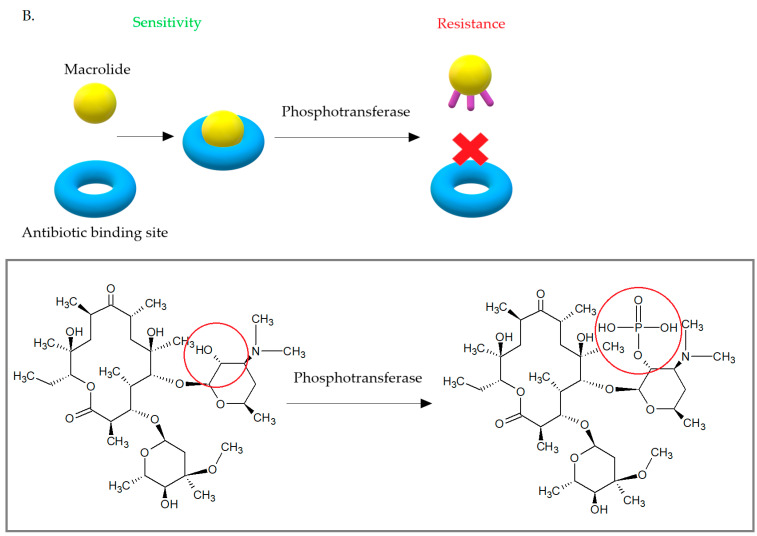
(**A**) Bacterial esterases cause hydrolysis of the erythromycin lactone ring, which prevents it from binding to the antibiotic target site. (**B**) Phosphotransferases introduce phosphate to the 2′-hydroxyl group of desosamine, which interferes with the interaction of the antibiotic with A2058.

Some *S. aureus* strains have developed the ability to enzymatically inactivate macrolides (Figure 11). Due to the low incidence, this mechanism is not important. Enzymatic inactivation of macrolides is associated with the presence of esterazes encoded by *empC*, *ereA*, and *ereB* [48]. The *ere* gene products cause hydrolytic inactivation of 14- and 15-membered macrolides and are the cause for high-level resistance to erythromycin [42,48]. The first esterase—*ereA* was isolated from *Escherichia coli* in 1984. The gene expression product was a protein of 44,8 kDa. The *ereB* gene was then isolated from another *E. coli* strain. The *ereA* gene is carried on the pIP1100 plasmid and the *ereB* gene was first identified on pIP1527 plasmid [42]. Both *ereA* and *ereB* encoded esterases hydrolyze the lactone ring of 14- and 15-membered macrolides, but show only 25% amino acid homology with each other. Macrolides with 16 carbons in the lactone ring and ketolides are not a substrate for these esterases [19,42,81].

Another enzymes—phosphotransferases—lead to changes in the structure of the 14-, 15-, and 16-membered lactone rings of macrolide antibiotics. Phosphotransferases introduce phosphate to the 2′-hydroxyl group of the macrolides amino sugar, which interferes with the interaction of the antibiotic with A2058. The phosphotransferases are usually coded on mobile genetic elements with other determinants of antibiotic resistance. These enzymes are encoded by *mphA* or *mphB* genes. They can be expressed by inducible (*mphA*) or constitutive (*mphB*) way [42]. Currently, there are seven macrolide-active phosphotransferases known: MphA, MphB, MphC, MphD, MphE, MphF, MphG. MphA, and MphB. These enzymes show 37% amino acid homology, but only MphB plays a role in the development of macrolide resistance in *S. aureus*, where it phosphorylates macrolides with 14- and 16-carbons in lactone ring. MphC was also isolated from clinical *S. aureus* strains and determined resistance to macrolides, but at low-level. The *mphC* gene is carried on pSN97 plasmid [42,82,83].

### 3.4. Another Mechanism of Resistance to Macrolides among S. aureus

Apart from the resistance mechanisms listed above, single cases of *S. aureus* macrolides resistance caused by other genes products have been reported. In some strains, the methylases ErmY and ErmF were discovered. Moreover, MefA protein leading to a low degree of resistance to macrolides with a 14-membered lactone ring was isolated from *S. aureus* [72]. There also have been few reports of chromosomal mutations leading to the development of resistance. Mutations in the *rplV* gene, which expresses the ribosomal protein L22, contributes to resistance to erythromycin, telithromycin, and Synercid. Mutations in the *rplD* chromosomal gene encoding the L4 protein result in resistance to erythromycin and spiromycin. A chromosomal mutation in the 50S ribosome subunit encoding region leads to change in the protein structure, which result in the development of erythromycin resistance [84]. Recently, Schwendener et al. discovered the novel *mef*(D), *msr*(F), and *msr*(H) macrolide resistance genes in resistance islands in *S. aureus*. The authors suggested that these islands may contribute to the spread of resistance among Staphylococcaceae species [85].

## 4. Conclusions

In times of increasing antibiotic resistance, it is important to understand the mechanisms of bacterial drug escape. Equally important is the rational use of antibiotics and the promotion of works leading to discoveries of new antimicrobial agents. Macrolides are antibiotics of first choice in the treatment of many infections, but the growing resistance to this group of drugs makes them diminish their value. Macrolide resistance in staphylococci is common and is associated with the presence of many molecular determinants. Furthermore, resistance to MLS_B_ is often associated with methicillin resistance which causes difficulties in treatment. Understanding the mechanisms of macrolide resistance is important in the context of research into new macrolide antibiotics, which could contribute to the fight against drug-resistant microorganisms.

## Figures and Tables

**Table 1:** Prevalence of cMLS_B_, iMLS_B_, and MS_B_ phenotypes (%) among MRSA and MSSA isolates [16,21,22,23,24,26,27,31,32,33,35].

Phenotype of Resistance to Methicillin	The Prevalence of cMLS_B_, iMLS_B_ and MS_B_ Phenotypes (%)			References
	cMLS_B_	iMLS_B_	MS_B_	
MRSA	73.7	18.4	7.9	[16]
MSSA	26.7	66.6	6.7	
MRSA	83	-	-	[21]
MSSA	-	82	-	
MRSA	-	76.4	-	[22]
MSSA	0	4.2	0	
MRSA	30.2	33.8	11.6	[23]
MSSA	24.4	0	0	
MRSA	51.89	18.5	0	[24]
MSSA	17.6	5.9	5.9	
MRSA	0	20	0	[26]
MSSA	0	16	0	
MRSA	84.3	6.25	9.375	[27]
MSSA	66.66	33.33	0	
MRSA	-	-	-	[28]
MSSA	69	5.4	1.8	
MRSA	68.2	4.5	4.5	[31]
MSSA	10.8	10.8	5.4	
MRSA	46.1	7.4	26.3	[32]
MSSA	-	-	-	
MRSA	18.6	33	4.7	[33]
MSSA	-	-	-	
MRSA	5.22	0.65	5.88	[35]
MSSA	7.84	8.49	13.07	

-: no data.

**Table 2:** Distribution of *ermA*, *ermB*, and *ermC* genes (%) among MRSA and MSSA strains [21,22,23,24,25,26,27,28,29,30,33,34,36,37].

Phenotype of Resistance to Methicillin	The Prevalence of *erm* Genes (%)			References
	*ermA*	*ermB*	*ermC*	
MRSA	57.6	0	4.9	[21]
MSSA	5.6	0.7	20.1	
MRSA	58.8	11.7	70.5	[22]
MSSA	4.2	0	0	
MRSA	7.69	13.84	27.69	[23]
MSSA	9.6	14.3	80.9	
MRSA	18.5	55.6	51.9	[24]
MSSA	11.8	29.4	47.1	
MRSA	46.7	0	36.7	[25]
MSSA	-	-	-	
MRSA	83.3	16.7	41.7	[26]
MSSA	32.4	2.7	10.8	
MRSA	62.5	0	84.375	[27]
MSSA	0	0	66.66	
MRSA	19	0	30	[28]
MSSA	9	0	33	
MRSA	16.7	0	66.7	[29]
MSSA	9.5	33.3	47.6	
MRSA	39.5	0	16.9	[30]
MSSA	1.6	0	0.8	
MRSA	51.6	57.57	84.84	[33]
MSSA	-	-	-	
MRSA	11.1	38.9	87	[34]
MSSA	11.1	38.9	87	
MRSA	35.66	0	13.05	[36]
MSSA	8.01	0	4.24	
MRSA	53.8	0	30.8	[37]
MSSA	-	-	-	

-: no data.

## Data Availability

No new data were created or analyzed in this study. Data sharing is not applicable to this article.

## Abbreviations

The following abbreviations are used in this manuscript:

A	a-site tRNA
ABC	ATP-binding-cassette
ABC-f	ATP-binding-cassette family
CA-MRSA	community-associated methicillin resistance *Staphylococcus aureus*
CLSI	Clinical and Laboratory Standards Institute
cMLS_B_	constitutive macrolides, lincosamides and streptogramin B phenotype
E	e-site tRNA
Erm	erythromycin resistance methylase
EUCAST	European Committee on Antimicrobial Susceptibility Testing
FDA	Food and Drug Administration
HA-MRSA	hospital-acquired methicillin resistance *Staphylococcus aureus*
hVISA	heterogeneous vancomycin intermediate-resistance *Staphylococcus aureus*
iMLSB	inductive macrolides, lincosamides, and streptogramin B phenotype
LA-MRSA	livestock-associated methicillin resistance *Staphylococcus aureus*
MAC-MRSA	Macrolide-resistant methicillin-resistant *Staphylococcus aureus*
MIC	minimal inhibitory concentration
MDR	multi-drug resistance
MLS_B_	macrolides, lincosamides and streptogramin B
MS_B_	phenotype of resistance to macrolides and streptogramin B
MRSA	methicillin resistance *Staphylococcus aureus*
MSD	membrane spanning domains
MSSA	methicillin sensitive *Staphylococcus aureus*
NBD	nucleotide binding domains
NPET	nascent peptide exit tunnel
ORF	open reading frame
P	*p*-site tRNA
PCT	phenyl transferase center
PRSA	penicillin resistance *Staphylococcus aureus*
RBS	ribosome binding site
TMD	transmembrane domains
WHO	World Health Organization
VISA	vancomycin intermediate-resistance *Staphylococcus aureus*
VRSA	vancomycin resistance *Staphylococcus aureus*
